# Breaking the Barrier: Pro-inflammatory Stool from Infants with CHD Triggers Barrier Dysfunction within Intestinal Organoids

**DOI:** 10.21203/rs.3.rs-10296197/v1

**Published:** 2026-07-31

**Authors:** Monalisha Elango, Kirtana Arikath, Haowen Qiu, Moorthi P. Ponnusamy, Adrian Black, Jeffrey D Salomon

**Affiliations:** 1 Department of Pediatrics, Child Health Research Institute, University of Nebraska Medical Center, Omaha, NE 68102, USA; 2 Department of Biochemistry and Molecular Biology, University of Nebraska Medical Center, Omaha, NE 68102, USA; 3 Nebraska Center for Biotechnology, University of Nebraska Lincoln, Lincoln, NE 68588, USA; 4 Eppley Institute for Research in Cancer and Allied Diseases, University of Nebraska Medical Center, Omaha, NE 68918, USA; 5 Department of Cellular and Integrative Physiology, University of Nebraska Medical Center, Omaha, NE 68102, USA

## Abstract

**Background:**

Infants with congenital heart disease (CHD) undergoing cardiopulmonary bypass (CPB) frequently develop gut injury and barrier dysfunction. The effects of the post-operative gut milieu on the intestinal epithelium remain poorly defined. Given ethical challenges in obtaining intestinal tissue from these patients, organoids offer a solution to study post-CPB intestinal changes.

**Methods:**

Porcine intestinal organoids were converted from basal-out to apical-out polarity to enable luminal exposure. Organoids were treated with pre-operative and post-operative fecal supernatants derived from a neonate with CHD undergoing CPB and a non-CHD surgical control. Microbial composition and metabolite profiles were analyzed, and epithelial responses were assessed following 72-hour exposure.

**Results:**

Apical-out organoids enabled direct luminal interrogation. Post-CPB stool exhibited enriched pro-inflammatory organisms and reduced microbial diversity, increased pro-inflammatory eicosanoids, and depletion of SCFA versus control. Post-CPB fecal supernatant induced epithelial barrier dysfunction and injury with redistribution of Claudin-2 and Claudin-3, and reduced fatty acid binding protein 2. There was altered PGE2 signaling including upregulation of PGE2 synthase, downregulation of prostaglandin dehydrogenase, and altered EP2 receptor localization.

**Conclusion:**

Post-operative stool following CPB contributes to epithelial injury and maladaptive responses characterized by PGE2-axis remodeling, tight junction reorganization, and loss of mature enterocyte features. This model of CHD stool exposure in organoids provides a robust translational platform for mechanistic studies and therapeutic targeting of gut injury following CPB.

## Introduction

Congenital heart disease (CHD) is among the most common birth defects, affecting roughly 8–10 per 1,000 live births worldwide and remaining a major cause of infant morbidity and mortality despite advances in diagnosis and surgery^1-5^. These patients have a disproportionate burden of gastrointestinal complications, notably feeding intolerance, growth failure, and necrotizing enterocolitis (NEC)^6-11^. Children with CHD have the second-highest incidence of NEC, which carries a mortality ranging from 10-30%^8,12,13^.

Emerging data implicate the gut microbiome as a key mediator of this heart–gut axis. Infants with CHD show altered gut microbial communities with reduced diversity and shifts in dominant taxa compared with healthy controls, patterns that often worsen perioperatively and contribute to the post-operative gut injury commonly seen in this patient population^7,11,14-17^. Children with CHD also show increases in pro-inflammatory eicosanoid profiles, specifically prostaglandin E2 (PGE2). In a recent cohort of infants with CHD undergoing cardiac surgery, postoperative samples were enriched for pathobionts such as *Escherichia–Shigella* and *Enterococcus*, and preoperative Enterococcus dominance was observed in the single infant who developed NEC^18,19^. These findings support a model in which CHD-related hypoperfusion, and medical/surgical exposures perturb the gut ecosystem, amplify mucosal and systemic inflammation, and contribute to adverse outcomes^20^.

Three-dimensional gut organoids offer an experimental platform to interrogate these mechanisms^21^. Intestinal organoids derived from animal models self-organize into structures that recapitulate key features of native intestine, including crypt–villus–like organization, multilineage differentiation, and epithelial barrier function^22-24^. These cultures preserve polarity and respond to luminal and basolateral cues more faithfully than traditional monolayers. Gut organoids are suited for exposure-related studies relevant to CHD, including inflammatory mediators, hypoxia, and therapeutic agents^25,26^. Intestinal organoids have been used to define pathogen-specific effects on epithelial damage, tight-junction integrity, and innate immune signaling^27^.

Within the CHD context, these platforms enable an opportunity to link clinical observations of dysbiosis and gut injury with mechanistic experimentation^11,17,28^. The ability to expose intestinal organoids to CHD-relevant conditions such as hypoxia, inflammatory metabolites, or stool from CHD patients offers the potential to interrogate how these conditions alter epithelial barrier function and epithelial health. Specific markers that have been identified to correlate with gut barrier function and integrity include claudin-2, claudin-3, and intestinal fatty acid binding protein (FABP2). Additionally, PGE2 has been shown to induce gut barrier dysfunction via the EP2 receptor^29-31^.

In this study, we evaluated the response of infant porcine-derived intestinal organoids to pre-operative and post-operative stool supernatants from neonates with and without CHD and assessed epithelial integrity and markers of barrier function. This study revealed that exposure to CHD patient supernatants led to a greater increase in markers of gut barrier dysfunction, including alterations in claudin-2, claudin-3, intestinal fatty acid binding protein (FABP2), and PGE2 signaling^29-31^, findings that parallel changes seen in CHD patients^11,17,28^. Thus, this study establishes infant pig organoids as a model for studying the connection between gut dysbiosis, inflammatory milieu, and barrier dysfunction to inform intestine-targeted interventions aimed at improving long-term health in children with CHD.

## Methods

### Animal Organoid Development

The current animal study was approved by the University of Nebraska Medical Center’s Institutional Animal Care and Use Committee, IACUC # 23-070-12-FC. All methods were performed in accordance with the Guide for the Care and Use of Laboratory Animals and the ARRIVE guidelines. Infant pigs between 5-6 weeks post-gestation, weighing 8-10 kg, were obtained from Oak Hill Genetics and acclimated to the housing facilities on the UNMC campus. These pigs were weaned off their mother’s breastmilk for 3 weeks and transitioned to feed. Antimicrobial-free feed selection was essential for this project. These animals were part of a larger study evaluating the gut effects and systemic inflammation of CPB. To create organoids, intestinal tissue was collected while the animals were still alive under general anesthesia with isoflurane and immediately placed in ice-cold PBS. Crypt isolation from jejunal tissue samples was performed within four hours. Following tissue collection, euthanasia was performed under anesthesia with a lethal pentobarbital dose injected directly into the heart.

Pig jejunal crypts isolation and culture as organoids was as described^32^. The mucosal layer of a 1-2 cm^2^ piece of jejunum was gently scraped using a sterile glass slide, then washed with PBS. The tissue was cut into ~0.5 × 0.5 cm segments and incubated in PBS containing 30 mM EDTA and 1 mM DTT. Tissue fragments were transferred to crypt washing buffer (54.9 mM D-Sorbitol + 43.4 mM Sucrose) and shaken vigorously for 2 min to release crypts. The suspension was passed through a 100 μm cell strainer and centrifuged at 200 × g for 2 min and repeated twice. The resulting pellet was resuspended in 200 μL Advanced DMEM/F12. Crypts were counted and embedded in ice-cold RGF Cultrex Type 2 BME with 5 crypts/μL and plated in 10-20 μL domes. After polymerization at 37°C for 30 min, domes were overlaid with organoid medium (Advanced DMEM/F12[Cat# 12634-010, Gibco], 1:100- N2 [Cat# 17502048 Gibco(100x)], 1:50-B27[Cat# A14867-01, Gibco], 1%L-Glutamine[Cat# 25-005-CI, CORNING], 1:100-HEPES(1M)[Cat# 25-060-CI, CORNING], 1% Penstrep(100x)[Cat# SV30079.01, R&D Systems], 10% Noggin[Conditioned medium], 10% R-Spondin [Conditioned medium], 50% WNT-3A [Conditioned medium], 1mM NAC[Cat# A9165, Sigma], 50mM EGF[Cat# 78136, STEMCELL Technologies], Primocin[Cat# ant-pm-1, Invivogen], 10mM NIC[Cat# N0636, Sigma], 10 μM SB202190[cat# S7067, Sigma], 0.5 μM A83-10[ Cat# 2939, Tocris Bioscience], 2.5 μM CHIR99021[Cat# SML1046], and 10 μM Y-27632 [cat# 10005583, Cayman chemical], and maintained at 37°C in a humidified 5% CO_2_ incubator. After two days, the medium containing Y-27632 was replaced with fresh complete organoid medium without Y-27632. The media was changed every 3 days. Passage was performed every 6 days. The organoids used in this study were at passage number 5 or below.

### Patient Stool Samples Collection and Storage

This study was approved by the institutional review board of the University of Nebraska Medical Center, IRB # IRB # 0070-20-FB. Informed Consent was obtained from the legal guardian prior to samples being collected and conformed to the principles in the Declaration of Helsinki. Stool samples from pediatric patients: a 2-week-old neonate with CHD undergoing a first stage palliation Norwood/BTT shunt surgery with cardiopulmonary bypass (CPB) and a 2-month-old comparison without CHD undergoing craniosynostosis repair surgery. One pre-op and one post-op sample were used in the study. These stool samples were part of a larger study and representative of the two cohorts, where microbiomic and metabolic analysis was already published on these^11,17^. Pre-operative stool was collected via spontaneous bowel movement using the Easy Sampler^®^ collection kit (ALPCO Diagnostics, Cat#58-EZSampler) and stored at −17 to −20°C, or using sterile FecalSwab^™^ (Copan Diagnostics, Cat#4CO24S) in the operating room. Post-operative stool was collected 2-5 days after surgery using the Easy Sampler^®^ collection kit. Stool samples were transferred and stored in a −80°C freezer.

### Stool Microbiome and Metabolite Profiling

DNA was extracted using the Qiagen PowerFecal DNA Extraction kit and purity quantified using the NanoDrop^™^ One (Thermo Fischer Scientific). Shotgun metagenomic sequencing libraries were prepared from extracted fecal DNA using the Illumina DNA Prep kit and sequenced on the Illumina NovaSeq 6000 platform generating 2 × 150 bp paired end reads. Raw sequencing reads were processed through the bioBakery 3 pipeline^33^. Taxonomic profiling was performed using MetaPhlan 4 generating relative abundance tables and were imported into R-package (v4.3) for alpha diversity analysis^34^. The datasets generated and/or analyzed during the current study are available in the NCBI Sequence Read Archive under BioProject ID PRJNA1497280.

Short-chain fatty acid (SCFA) and eicosanoid metabolites were extracted from stool and subsequently quantified using ultra-performance liquid chromatography-tandem mass spectrometry (UPLC-MS/MS) per previously published protocols^11,28^ via the Shimadzu 8060NX mass spectrometer (Shimadzu Scientific Instruments, Columbia, MD). SCFA and eicosanoid levels were determined using the LabSolution software (version 5.99). Selected SCFA and Eicosanoids are represented, and graphs are created in GraphPad Prism. Since only one sample for pre-op and post-op time points, only descriptive analysis is provided.

### Basal Out to Apical-Out Organoid Polarity Inversion

ECM-embedded organoids of uniform size (150–250 μm diameter; day 3–5 post-passaging) were selected, and organoid health was confirmed by the presence of intact cystic and/or budding structures with minimal debris. Organoids develop in a basal-out (BO) configuration. For ECM removal and induction of apical-out (AO) polarity, GCDR (Gentle Cell Dissociation Reagent) [Cat# 13151-014, Gibco] and DMEM/F-12 were pre-cooled (4°C). The overlay medium was aspirated without disrupting the Matrigel domes and the domes were incubated with 1 mL ice-cold GCDR per well. A 1 mL pipette tip was rendered non-adherent by coating with Anti-Adherent Rinsing Solution [Cat# 07010, STEMCELL Technologies]. Domes were transferred into a pre-coated, anti-adherent 24-well plate containing 0.5 mL cold DMEM/F-12 per well. Organoids were resuspended in 0.5–1.0 mL pre-warmed complete organoid medium (supplemented with 10 μM Y-27632 for the first 24 h) and transferred to standard culture conditions (37°C, 5% CO_2_) for AO suspension culture. Medium exchanges were performed daily by removing 0.4 mL and replacing it with 0.4 mL warm complete organoid medium. By day 3, organoids exhibited outward-facing AO epithelial polarity, confirmed with immunofluorescent staining.

### Stool Exposure Study

Fecal supernatant (FS) was prepared from stool samples as follows: frozen stool was thawed on ice and weighed, then homogenized in ice-cold PBS at a 200%(w/v) ratio (e.g., 200 mg stool in 1 mL PBS) by vortexing for 1–2 min. The homogenate was centrifuged at 4,000 × g for 10 min at 4°C, and the supernatant was collected. The supernatant was further centrifuged at 10,000 × g for 10 min at 4°C and filtered through a 0.22 μm filter. The resulting FS was used for organoid exposure. Organoids were seeded at equal density into 24-well plates and assigned to the following treatment conditions: CPB preoperative FS (CPB-Pre-op), CPB postoperative FS (CPB-Post-op), comparison preoperative FS (Comp-Pre-op), comparison postoperative FS (Comp-Post-op). For exposure, culture medium was replaced with complete medium supplemented with FS at a final dilution of 1:100 (v/v). On day 3 of exposure, organoids were washed once with PBS, fixed in methanol, embedded in HistoGel, and cryosectioned for staining. The exposure experiments were performed twice with the same stool sample.

### Immunofluorescence and Immunohistochemical Staining

BO and AO organoids were immune-stained with structural proteins using antibodies against ZO-1 (Cat# bs-1329R) and E-Cadherin (Cat# 60335-1-Ig). Stool exposure organoids were immune-stained for inflammatory markers, Claudin 2 (Cat # MBS820337), Claudin 3 (Cat # 34-1700), FABP2 (Cat # MBS2032817), Prostaglandin EP2 receptor (Cat # MA5-35750), Prostaglandin E2 synthase (PTGES) (Cat # 10881-1-AP), and Prostaglandin dehydrogenase (15-PGDH) (Cat # MA5-38132). Images were acquired with an Echo Revolve R4 microscope in inverted mode (Discover Echo), employing 20x PLAN X Apo objectives with a numerical aperture of 0.80 and a working distance of 0.6mm.

### Image and Statistical Analysis

Immunofluorescence images were analyzed using Fiji/ImageJ (NIH). Images for each marker were captured under identical microscope settings across all groups and quantified from raw files following a standardized workflow. Only intact, in-focus organoid sections without folds, tears, or overlap were included. After channel separation, a region of interest covering the epithelial compartment of each organoid was manually outlined using DAPI staining and tissue morphology as references, while excluding the luminal space and the surrounding background. Mean fluorescence intensity and integrated density were measured for each Region of Interest, with background fluorescence obtained from nearby cell-free areas. For each marker, identical thresholding and measurement settings were applied. Data are expressed as relative fluorescence normalized to the corresponding control pre-operative samples. Quantification for junction-associated markers was limited to the epithelial ring. Quantification was conducted in Fiji/ImageJ using corrected fluorescence methods.

Quantitative data were analyzed using one-way analysis of variance (one-way ANOVA) in GraphPad Prism version 10.6.2 (892) to compare differences among the experimental groups, including Control pre-operative, Control post-operative, CPB pre-operative, and CPB post-operative conditions. When the ANOVA was significant, multiple group comparisons were performed using Tukey’s post hoc test. Data are presented as mean ± SD, and a P value < 0.05 was considered statistically significant. The microbial and metabolite data of the stool samples are presented as descriptive since the same stool samples from both patients were used in the duplicate stool exposure experimentation.

### Patient and Public Involvement

Patients and the public were not involved in the design of this study. It was not possible to involve patients or the public in the design, conduct, or reporting, or dissemination plans of our research.

## Results:

### BO to AO polarity conversion enables direct luminal exposure

Infant porcine jejunal crypts were used to produce BO to AO configuration for luminal challenge experiments. This process aligns with the well-established principle that matrix-embedded intestinal organoids receive basal cues via integrins/ECM, maintaining a basal-out, apical-in organization. Removing the ECM in suspension allows polarity inversion and apical surface eversion^32,35,36^.

**Figure 1.**
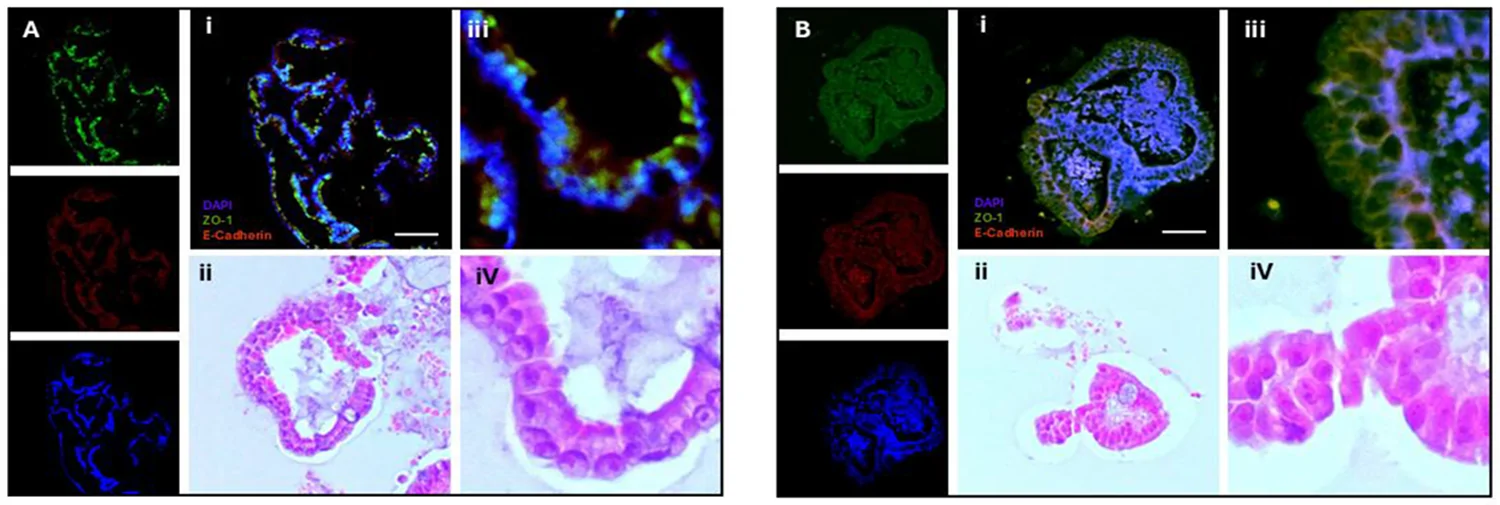
Structural validation of Basal-out and Apical-out porcine jejunal organoids. **(A)** Basal-out and **(B)** Apical-out porcine jejunal organoids were assessed by immunofluorescence and H&E staining. Single-channel images show **ZO-1** in green, **E-cadherin** in red, and **DAPI** in blue. Merged images **(i)** and enlarged views **(iii)** demonstrate epithelial organization and junctional marker localization. H&E images **(ii, iv)** confirm preservation of epithelial morphology after polarity conversion. Basal-out organoids show apical ZO-1 localization toward the internal lumen, whereas apical-out organoids display outward-facing epithelial polarity suitable for direct luminal exposure studies. Scale bar: 200 μm.

Polarity conversion and preservation of epithelial organization were successfully achieved in porcine intestinal organoid (Figure 1). ZO-1 and E-cadherin staining in the BO→AO panels continued to show organized epithelial structure and junctional integrity after the conversion (Figure 1A, 1B). The ZO-1 (green) lining is on the inside, and the ZO-1 marker appears facing outward, confirming the suitability of the AO platform for luminal challenge studies (Figure 1A,1B). In intestinal organoids, ZO-1 highlights the apical tight junction domain, while E-cadherin indicates epithelial cell–cell adhesion. The retention of these polarity markers after inversion is a standard sign that AO conversion has occurred without significant structural failure. Apical out organoids have the apical membrane facing outward (Figure 1B (i)), exposed directly to the surrounding medium, which allows soluble luminal factors to interact with the epithelium in a way that more accurately mimics in vivo host–microbe and diet–epithelium interactions^35-37^. Hematoxylin and Eosin-stained sections are shown at lower magnification in (Figure 1A,1B (ii)) and at higher magnification in (Figure 1A,1B (iv)) to demonstrate preservation of organoid epithelial architecture after polarity conversion. Overall, the workflow and marker staining confirm that porcine jejunal organoids were successfully converted from basal-out to apical-out polarity, creating a luminally accessible epithelial platform for testing stool-derived factors related to CHD/CPB-associated intestinal injury.

### CHD post-operative stool exhibits dysbiosis and a pro-inflammatory metabolite profile

**Figure 2.**
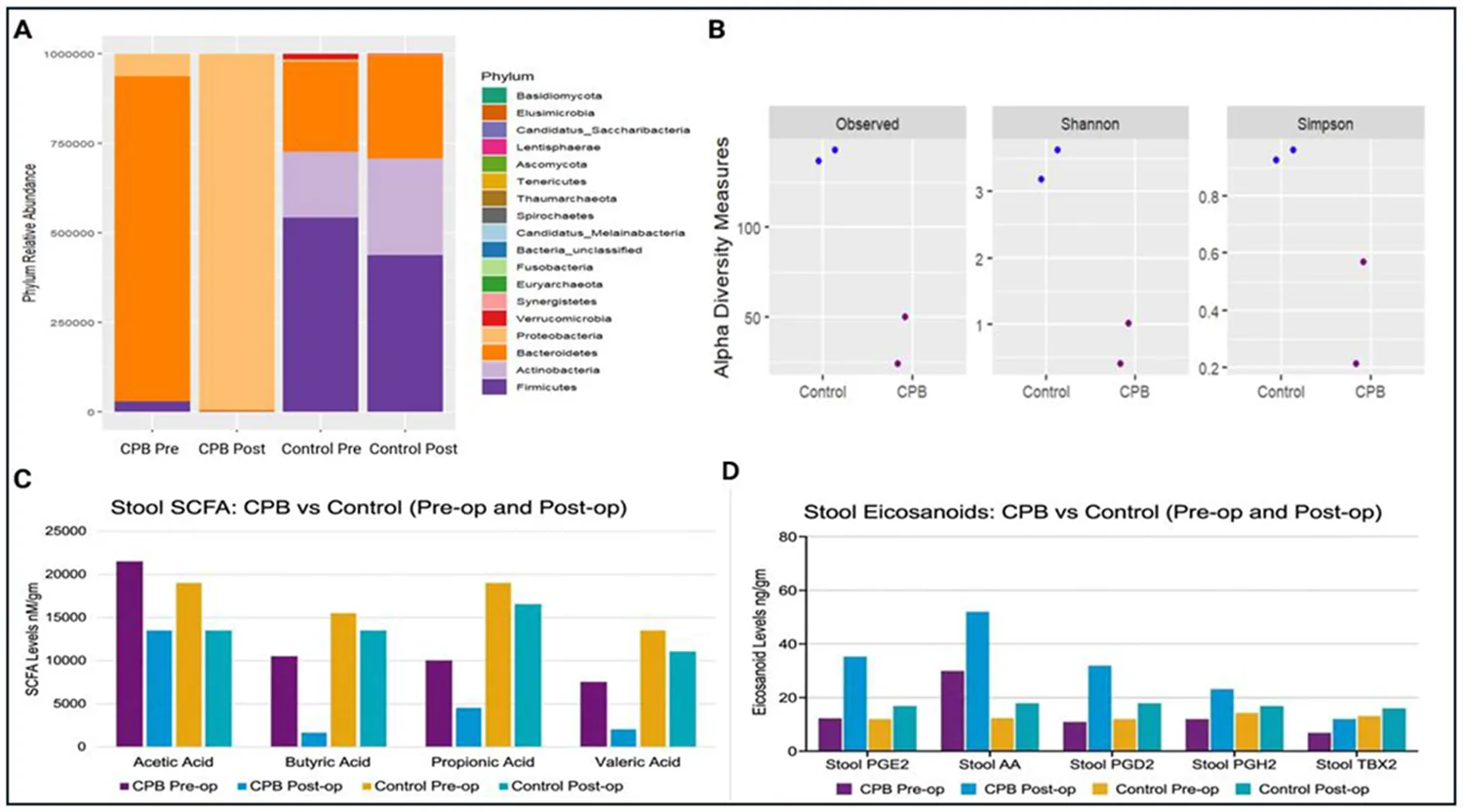
CHD/CPB-associated stool shows altered microbial composition and pro-inflammatory milieu. Stool samples from CPB pre-operative, CPB post-operative, Control pre-operative, and Control post-operative groups were analyzed for microbial and metabolite profiles. (A) Stacked bar plots showing the relative abundance of bacterial phyla and differences in overall phylum-level microbial composition across the four conditions. (B) Alpha diversity of the stool microbiota, assessed by observed richness, Shannon index, and Simpson index. Each point represents an individual sample and shows reduced microbial diversity in CPB compared with Control samples. (C) Bar graphs showing stool short-chain fatty acid (SCFA) levels, including acetic acid, butyric acid, propionic acid, and valeric acid, across the four conditions. (D) Bar graphs showing stool eicosanoid levels, including PGE2, AA, PGD2, PGH2, and TBX2, across the four conditions.

The stool samples selected are from a larger data set and are representative, showing the differences in the microbiome and metabolite profiles of the CPB group and comparison group in a larger published study^11,17^. The CHD sample had lower microbial diversity and higher levels of pro-inflammatory bacteria compared to the non-CHD subject (Figure 2A,2B). This aligns with previous pediatric CHD research, which shows baseline dysbiosis that worsens after CPB, increasing genera associated with pathobionts, like *E. coli* and *Enterococcus*^11,17,18^.

Metabolically, the stool samples exhibited largely similar pre-operative eicosanoid profiles across the two subjects. However, post-operative stool in CHD showed a selective increase in pro-inflammatory eicosanoids and a significant decrease in short-chain fatty acids (SCFAs) (Figure 2C,2D). This pattern aligns closely with published data from human and piglet CPB studies, in which bypass correlates with increased stool eicosanoids, including a twofold rise in PGE_2_-related signals, along with notable reductions in acetic, butyric, and propionic acids^11,28^. Overall, these results characterize the CHD post-operative stool to have evidence of dysbiosis, increased pro-inflammatory eicosanoids, and decreased gut protective SCFA metabolites.

### CPB-associated FS drives tight-junction remodeling and enterocyte injury

**Figure 3.**
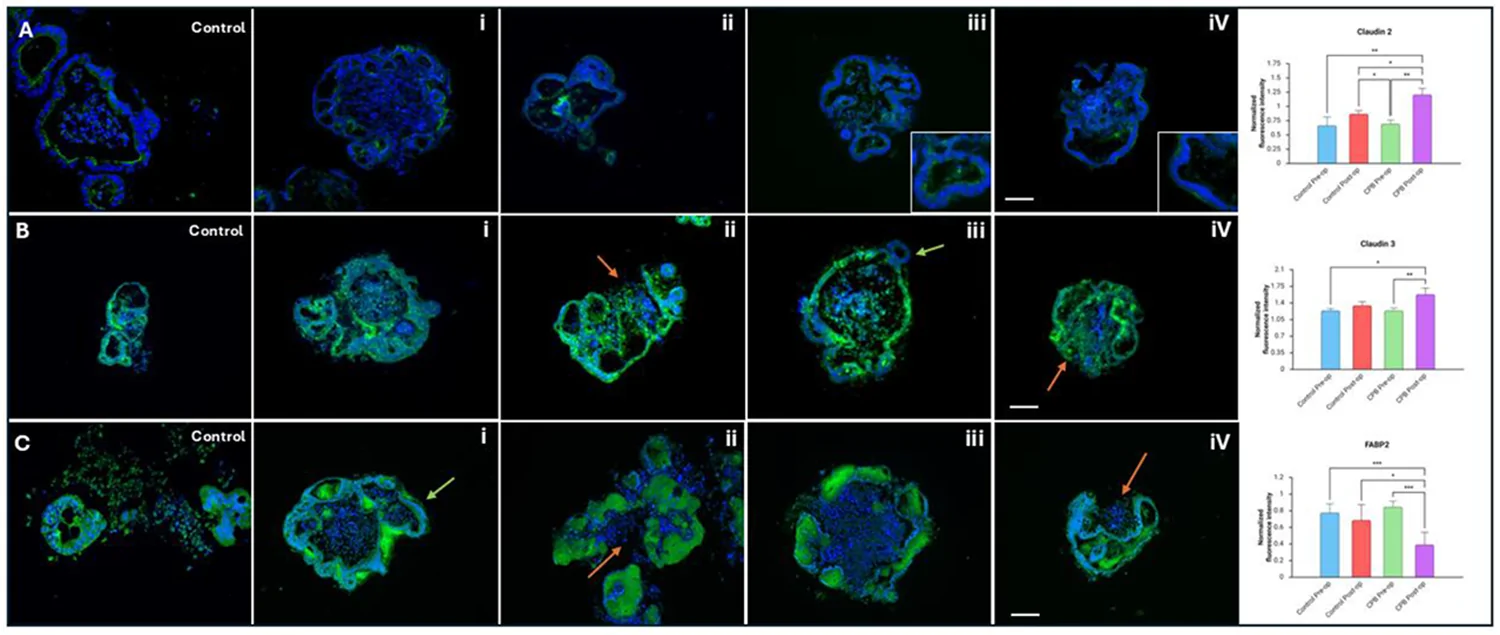
Stool exposure alters Claudin-2, Claudin-3, and FABP2 expression in porcine intestinal organoids. (Control) Images are non-exposed organoids. Organoids were exposed to stool samples from (i) Control Pre-op, (ii) Control Post-op, (iii) CPB Pre-op, and (iv) CPB Post-op groups. (A) Representative immunofluorescence images and quantification of Claudin-2. Claudin-2 is shown in green, and nuclei are counterstained with DAPI (blue). Insets highlight representative regions of staining; in CPB Pre-op, the signal appears as a more internal ring, whereas in CPB Post-op, it appears as a more external ring. The bar graph summarizes normalized fluorescence intensity across the four conditions. (B) Representative immunofluorescence images and quantification of Claudin-3. Claudin-3 is shown in green, and nuclei are counterstained with DAPI (blue). (C) Representative immunofluorescence images and quantification of FABP2. FABP2 is shown in green, and nuclei are counterstained with DAPI (blue). The green arrow shows the healthy crypt and Villi domains, and the orange arrow shows the damage to the organoid structure. Data are presented as mean ± SD and analyzed by one-way ANOVA. Quantification was performed from independent images per group as follows: Claudin-2, n = 4, 5, 4, and 4; Claudin-3, n = 4, 4, 4, and 5; FABP2, n = 6, 7, 3, and 8, corresponding to groups i–iv, respectively. Scale bar: 250 μm.

In AO porcine intestinal organoids exposed for 3 days to human fecal supernatant, the tight junction response was characterized by marker-specific remodeling rather than uniform junction loss. Claudin-3, a barrier sealing claudin in the epithelium, was significantly increased in the CPB post-operative group compared to other groups, while Claudin-2, a pore-forming tight junction protein that creates paracellular channels for small cations and water and is widely linked to “leaky” epithelial states during intestinal inflammation^38,39^, was significantly higher in the CPB post-op compared to other groups (Figure 3A,3B). For Claudin-3, post-operative samples showed structural disruption, and for Claudin-2, the staining pattern shifted from a more internal ring in pre-op samples to a more external distribution in post-op samples, indicating altered junctional organization (Figure 3A,3B).

FABP2/I-FABP signal was significantly reduced in CPB post-op human FS-exposed AO organoids compared to pre-op samples (Figure 3C). Additionally, CPB post-op organoids show the most significant structural disruption (Figure 3B,3C). This finding confirms that FABP2 is enriched in absorptive villus enterocytes, with little to no expression in crypt cells.

### Post-operative FS induces coordinated remodeling of the epithelial prostaglandin E2 signaling axis

**Figure 4.**
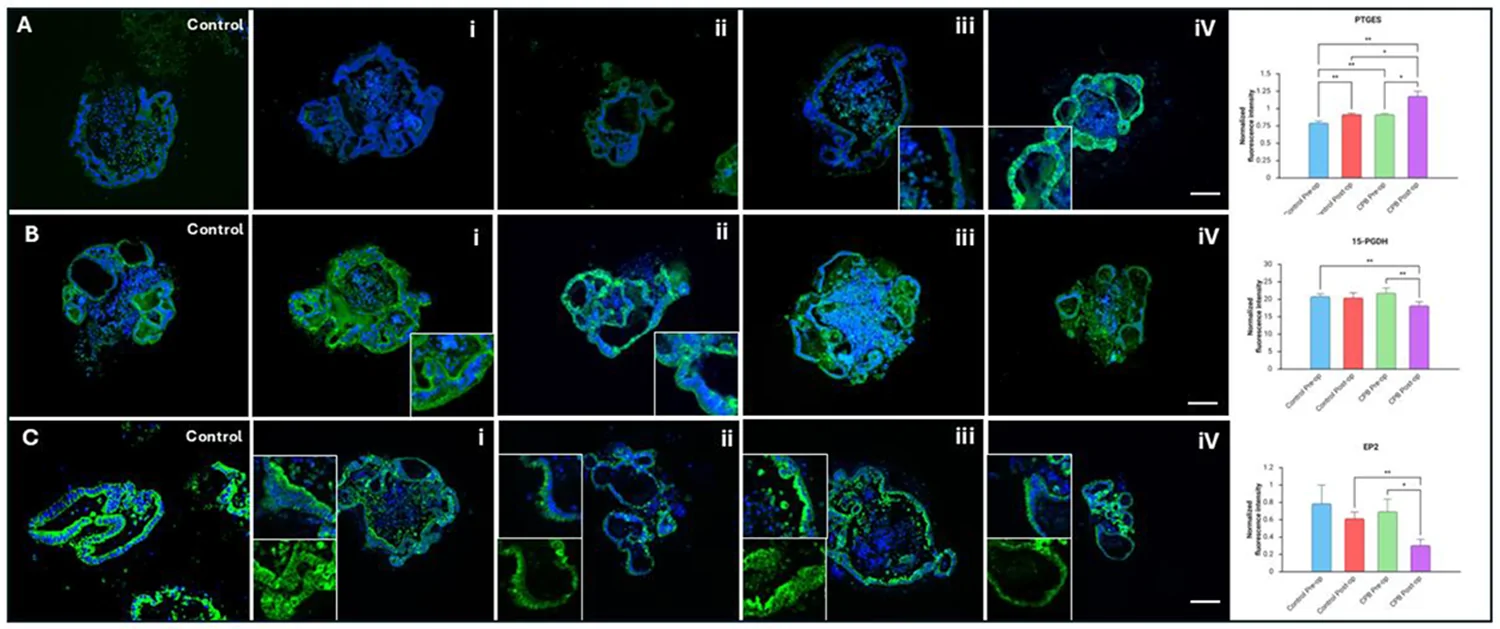
Stool exposure alters PTGES, 15-PGDH, and EP2 expression in porcine intestinal organoids. (Control) Images are non-exposed organoids. Organoids were exposed to stool samples from (i) Control Pre-op, (ii) Control Post-op, (iii) CPB Pre-op, and (iv) CPB Post-op groups. (A) Representative immunofluorescence images and quantification of PTGES. PTGES is shown in green, and nuclei are counterstained with DAPI (blue). Insets highlight representative regions of PTGES localization. The accompanying bar graph shows normalized fluorescence intensity across the four conditions. (B) Representative immunofluorescence images and quantification of 15-PGDH. 15-PGDH is shown in green, and nuclei are counterstained with DAPI (blue). Insets highlight representative staining patterns, and the accompanying bar graph shows normalized fluorescence intensity across the four conditions. (C) Representative immunofluorescence images and quantification of EP2. EP2 is shown in green, and nuclei are counterstained with DAPI (blue). Insets highlight representative regions of epithelial localization, and the accompanying bar graph shows normalized fluorescence intensity across the four conditions. Data are presented as mean ± SD and were analyzed by one-way ANOVA. Quantification was performed from independent images per group as follows: PTGES, n = 4 per group; 15-PGDH, n = 4, 3, 5, and 8; EP2, n = 4, 4, 4, and 8, corresponding to groups i–iv, respectively. Scale bar: 250 μm.

Exposure of AO porcine intestinal organoids to human FS for 3 days triggered a coordinated, condition-dependent change in markers involved in epithelial PGE2 production, degradation, and receptor signaling. PTGES/mPGES-1 increased after post-operative exposure in both groups, with the most notable increase observed in the CPB post-operative condition compared to the CPB pre-operative state (Figure 4A). Conversely, the 15-PGDH/HPGD decreased in CPB post-operative organoids relative to pre-operative ones (Figure 4B), and EP2 staining also decreased in the CPB post-operative condition, suggesting enterocyte injury and receptor-level remodeling rather than a static prostaglandin response (Figure 4C). These marker changes support a shift toward altered epithelial PGE2 signaling under post-operative luminal conditions, especially in the CPB postoperative environment. In Figure 4, PGE2 and receptor signaling show redistribution in the CPB stool exposed organoids, where the pre-op stool exposure signal appears as a more internal ring, and the post-op stool exposure signal becomes more external, along with structural disruption in the organoids. Overall, a remodeling of epithelial EP2-associated PGE2 signaling in the CPB post-operative luminal environment coincides with barrier disruption and enterocyte injury.

## Discussion

This study supports a model in which the post-operative CHD/CPB luminal environment is sufficient to trigger a coordinated epithelial injury-response program in intestinal organoids. The main findings show the convergence of several biologically related indicators: a shift in the prostaglandin axis, remodeling of tight-junction composition and organization, and loss of an enterocyte-associated marker following exposure to stool-derived soluble factors from CHD/CPB. That overall pattern aligns with the broader pediatric CHD literature, where cardiopulmonary bypass has been associated with perioperative dysbiosis, impaired barrier function, reduced short-chain fatty acids, and gastrointestinal morbidity^19,20^. This model can be used to pinpoint specific metabolite and organism exposure based upon analysis of patients within this population and further enhance our understanding of specific drivers of the gut injury observed following pediatric cardiac surgery with CPB.

The stool data provide the biological context for these epithelial responses. The CHD stool sample showed reduced diversity, increased pro-inflammatory eicosanoids, and lower SCFAs. This aligns with previous CHD/CPB studies^11,18,19^. Mechanistically, this is important because inflammatory lipid mediators can alter epithelial signaling, junctional behavior, promote immune cell differentiation, and induce apoptosis, while SCFA depletion eliminates a key trophic input that normally supports epithelial metabolism, barrier integrity, and promotes anti-inflammatory immune cell production^19,20,40^.

A major strength of the study is the use of the AO organoid format. By reversing epithelial polarity, the model exposes the apical surface directly to luminal contents and avoids the technical variability of microinjection. This is important because the biological question specifically concerns whether stool-derived luminal signals can act directly on the intestinal epithelium. The maintained organization of ZO-1 and E-cadherin after converting from BO to AO polarization supports that the platform remains structurally suitable for exposure studies. Additionally, porcine AO intestinal organoids are increasingly recognized as a translational system for studying apical nutrient uptake, barrier responses, and host-luminal interactions^32,41^.

The Claudin-2/Claudin-3 pattern indicates the tight-junction remodeling. Claudin-2 is a pore-forming claudin linked to increased cation and water flux in inflamed, leakier epithelia, while claudin biology in the intestine is strongly influenced by spatial expression, inflammatory trafficking, and repair-related redistribution. Claudin-3, on the other hand, is generally associated with barrier maturity and integrity, and changes in its levels or location can reflect an epithelial state shift rather than complete barrier failure. The decrease in FABP2 adds a second important layer because it marks mature absorptive enterocytes and declines when these cells are injured. This finding also aligns with the clinical CPB literature, where circulating I-FABP increases after infant cardiothoracic surgery, indicating enterocyte injury^38,42-46^. During intestinal ischemia-reperfusion, cytosolic I-FABP decreases in mature enterocytes, while staining shows this protein appears in the subepithelial space and luminal debris. This indicates leakage of intracellular FABP2 from damaged epithelial cells during shedding and early barrier repair^45^. More broadly, I-FABP is recognized as a sensitive marker of small-intestinal ischemic epithelial injury because it is released into the circulation when enterocytes are damaged. Limitations of this study are in the measures of enterocyte injury and death. We focused on a known intracellular marker of enterocyte injury, FABP2. In human studies, when this is measured in circulation, it is indicative of cell injury, and plasma levels correlate with mucosal injury in both animal and human ischemia-reperfusion models^45,47^. Considering our exposure results, the reduced intracellular FABP2 signal in CPB-exposed organoids indicates enterocyte injury and altered epithelial condition.

The prostaglandin findings are internally consistent and likely represent the most mechanistically informative part of the dataset. PTGES upregulation, along with reduced 15-PGDH in the post-operative condition of CPB, suggests increased effective epithelial PGE2 levels. In the intestine, mPGES-1 is an inducible enzyme involved in inflammation, while 15-PGDH is the main enzyme responsible for breaking down PGE2, limiting its accumulation locally. Reduced 15-PGDH in inflamed mucosa has been linked to TNFα-dependent suppression. Simultaneously, PGE2 is not merely a pro-inflammatory bystander in the gut; it also plays a role in epithelial adaptation and wound healing. In cases of intestinal injury, prostaglandins can promote reprogramming rather than solely cause damage^48,49^.

PTGES encodes microsomal prostaglandin E synthase-1, the inducible enzyme that converts PGH2 to PGE2 and plays a major role in the production of inflammatory PGE2. Studies on experimental colitis identify mPGES-1 as a key component of intestinal inflammatory responses, and broader gastrointestinal prostaglandin research supports PGE2 as an important mediator of mucosal response that is receptor-dependent. By contrast, 15-PGDH is the dominant prostaglandin-degrading enzyme in intestinal epithelium and reduced 15-PGDH expression has been documented in inflamed intestinal mucosa, including settings in which TNF-α suppresses epithelial 15-PGDH expression^48,50^. Research in Caco-2 cells demonstrated that PGE2 signaling through EP1/EP2 can lead to apical junctional complex disassembly and redistribution of junction-associated proteins^51,52^. The EP2 receptor has been shown to induce gut inflammation and gut barrier dysfunction, while the EP4 receptor has been noted to promote protection, injury response, and inflammatory remodeling^53,54^. The higher PTGES, together with lower 15-PGDH seen in the CPB post-operative condition, is interpreted as increased effective epithelial PGE2, rather than as unrelated fluctuations in individual markers^48,53^. Therefore, CPB-associated post-operative stool exposure affects EP2 receptor expression and/or localization along with broader PGE2-axis remodeling, indicating an injury-responsive epithelial signaling state rather than a static homeostatic prostaglandin system. Additionally, it is unknown if transcription profiles of the organoids are retained from the original porcine tissue. Future experiments to evaluate this will increase the clinical translation of this model for other exposures.

While stool samples from only one patient in each clinical group were used in this exposure experiment, each patient acts as their own pre-op versus post-op control. Rather than introduce multiple patient samples into this study, which might have diluted the results, we used the same stool samples on multiple batches of organoids to confirm the response was reproducible. Future studies with fecal supernatants from multiple patients, or a combination of fecal slurries from multiple patients are necessary to show this is a phenotype shared across the entire patient population. The focus of these experiments was to demonstrate reproducibility of the organoid response and support this model can be used to study the gut injury phenomenon in this patient population.

**Figure 5.**
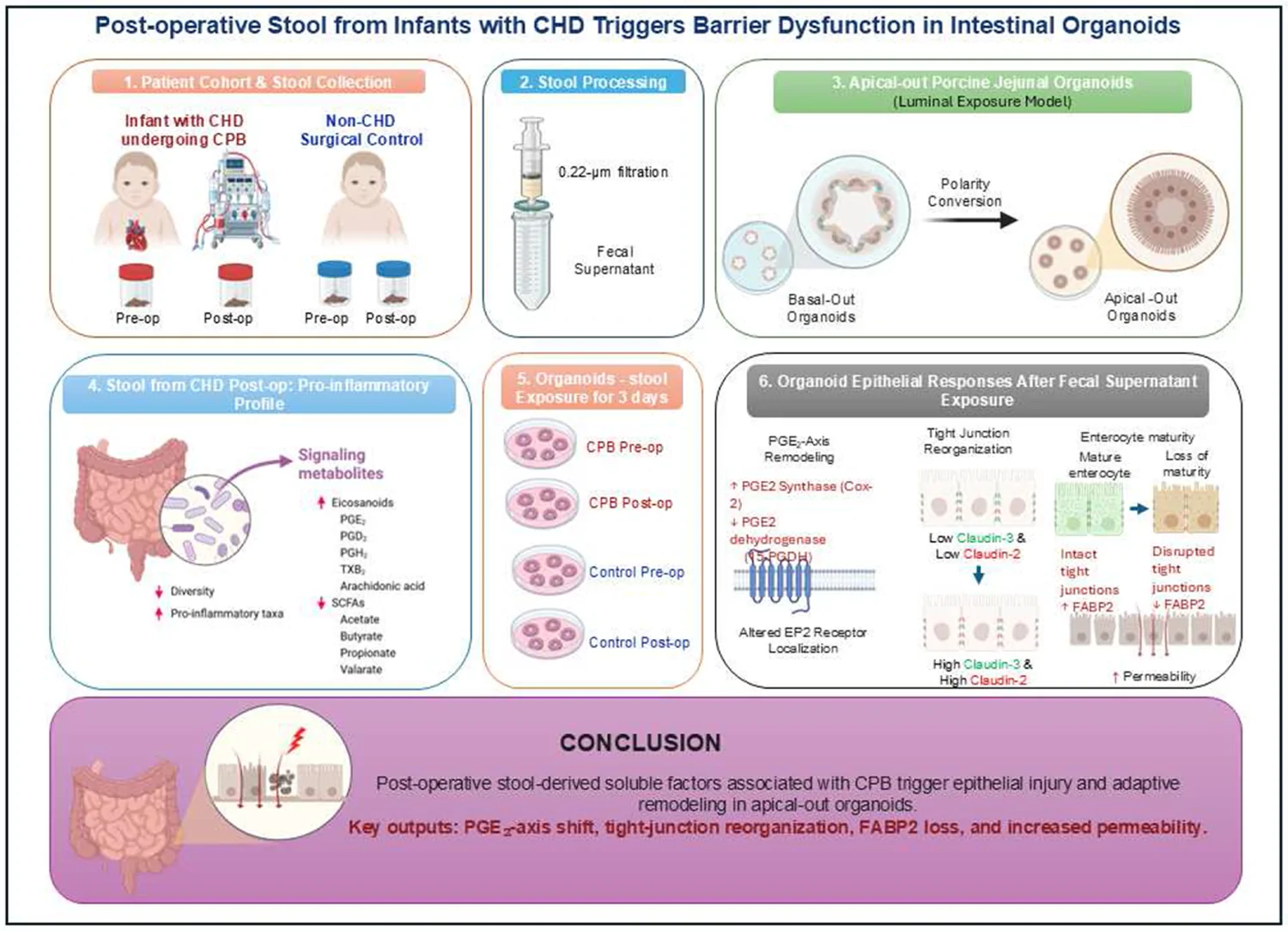
Schematic of the epithelial injury involving barrier dysfunction and altered PGE-2 axis. Exposure of pre-operative and post-operative stool from an infant with CHD with increased pro-inflammatory metabolites to apical-out infant porcine organoids results in evidence of barrier dysfunction and epithelial disruption. This coincides with alterations in the PGE-2 axis signaling, resulting in increased permeability and gut injury.

In summary, this study connects clinically relevant CHD/CPB stool dysbiosis to a specific epithelial response in a luminally accessible organoid system. The data support a model where post-operative CPB stool-derived soluble factors alter epithelial prostaglandin signaling, reorganize tight junctions, and reduce an enterocyte-associated differentiation/injury marker, with the most significant effects observed in the CPB post-operative condition (Figure 5). Taken together, these results suggest that the post-CPB fecal supernatant has a specific mixture of dysbiosis-related metabolites, inflammatory lipid mediators, and microbial products capable of altering epithelial signaling and structure toward an injury-susceptible phenotype. The prostaglandin axis seems to be one such response mechanism, while changes in claudin and FABP2 indicate that junctional architecture and enterocyte health are also being affected. This interpretation aligns with recent porcine CPB studies showing dysbiosis, altered stool metabolites, and disrupted claudin/FABP2-related intestinal barrier biology after bypass^20,28^ and sets the stage for specific metabolite or organism exposure experiments, including the use of organoids from infant cardiopulmonary bypass to recapitulate the inflammatory insult on intestinal tissue.

## Key Message:

### What is already known on this topic:

Organoids can be used to function as exposure experiments. Stool from infants with CHD contains a pro-inflammatory milieu.

### What this study adds:

Organoid exposure to stool from infants with CHD induces epithelial injury and barrier dysfunction. This injury and barrier disruption occurs, in part, through modification of the PGE2 axis

### How this study might affect research, practice, or policy:

Understanding the mechanisms by which PGE2 induces alterations in tight junction proteins and mediates cellular injury is essential. This study also promotes a potential therapeutic target to reduce gut injury and barrier dysfunction that is seen in children after cardiac surgery who show elevations in PGE2 and other pro-inflammatory milieu.
